# Professional identity measures for student health professionals – a systematic review of psychometric properties

**DOI:** 10.1186/s12909-019-1660-5

**Published:** 2019-08-13

**Authors:** Jordan Matthews, Andrea Bialocerkowski, Matthew Molineux

**Affiliations:** 1 Griffith Health, Griffith University, Queensland, Australia; 2 Discipline of Occupational Therapy, School of Allied Health Sciences, Griffith University, Queensland, Australia

**Keywords:** Professional identity measure, Health, Students, Psychometric, Methodological quality

## Abstract

**Background:**

Professional identity is critical to the safe and effective clinical practice of all health professions. University programs play an important role in the formation of professional identity of students, and so it essential to understand professional identity at this stage of students’ development. However, the majority of research into professional identity has been conducted using the qualitative paradigm so further quantitative analysis through the use of psychometrically-sound professional identity measures is required. This study aimed to identify professional identity measures used with university students enrolled in health programs and synthesise the evidence of their psychometric properties.

**Methods:**

The systematic review was conducted in two phases. Phase 1 involved searching five online databases for studies that used professional identity measures with student health professionals. These studies were assessed against a priori criteria for inclusion and a list of measures was identified. Phase 2 involved searching the same databases for psychometric evidence of the measures identified in Phase 1. The psychometric properties of each measure were compared against the Consensus-based standards for the Selection of Health Measurement Instruments (COSMIN) checklist. Data were narratively synthesised, and comparisons were made between measures.

**Results:**

Phase 1 identified eight professional identity measures. Phase 2 identified a total of 15 studies that evaluated the psychometric properties of at least one of the professional identity measures. There was a paucity of psychometric evidence for the measures. The revised Nurses’ Professional Values Scale and Macleod Clark Professional Identity Scale had the greatest volume of psychometric evidence. None of the measures fulfilled all criteria in the COSMIN checklist.

**Conclusion:**

There is a paucity of evidence underpinning the psychometric of professional identity measures. Evidence which uses these measures should be interpreted with caution. Further research is warranted to ensure that the results of quantitative professional identity studies are valid and reliable.

## Background

Professional identity is defined as “the attitudes, values, knowledge, beliefs and skills shared with others within a professional group” [1]. The development of professional identity has been noted as a continuous process that is influenced by several factors including experiences in practice and professional socialisation [2]. A significant amount of this development occurs during an individual’s time completing a university program. Professional identity is considered a dynamic phenomenon, which continues to evolve from university study into a health professionals’ work life [3, 4].

In a health setting, professional identity serves a great importance. It defines practice boundaries and assists in limiting role confusion particularly in multidisciplinary teams [5]. A lack of professional identity clarity has been found to have a significant impact on a profession’s perceived value and on a practitioner’s confidence in advocating for their professional opinions [6]. These difficulties also influence an educators’ confidence to instil appropriate professional knowledge and values in their students [7]. Further, an unclear professional identity can lead to larger issues within a profession in which practice become less paradigm-specific and increasingly focused on roles that ‘fill gaps’ of other professions [8].

The impact professional identity has on practice in health settings has resulted in an increased attention aimed at understanding the concept and its development. With university programs playing an important role in preparing students for the workforce by promoting professional identity development, qualitative research has explored this area. For example, Ashby, Adler and Herbert [2] explored the impact of various aspects of university curricula and their impacts on professional identity development, from a student perspective. Practice education and specific content related to the core values of the profession were perceived as the most influential on a student’s development of their professional identity. Engagement in group coaching has also been found to positively influence identity formation, particularly during transition from student to practitioner [4]. Other researchers have examined specific aspects of university programs, such as role-emerging placements, and found that this experience assisted students to develop a deeper understanding of self and provided clarity regarding the uniqueness of their profession [9]. The provision of constructive feedback and opportunities for meaningful interaction with a variety of members of a profession have been noted to benefit students’ professional behaviours [10], as has reflection [11] and reflective writing [12].

These findings have provided researchers with an understanding of the effect of different experiences on the development of professional identity from students’ perspectives. However, to develop strategies to strengthen professional identity requires support from quantitative evidence. Given the importance being placed on interprofessional education and its role in producing a collaborative, practice-ready health workforce [13], comparison of students’ professional identity between health professions, through quantitative methods, will be critical to ensure that future service provision remains unique despite increased shared learning in university curricula.

Quantitative research into professional identity requires tools that measure professional identity which are psychometrically sound, with acceptable levels of reliability and validity. Reliability is defined as “the extent to which scores… are the same for repeated measurement under several conditions”, while validity is defined as “the degree to which an instrument measures the construct(s) it purports to measure” [14]. Without adequate reliability, the results of a measure may be inconsistent upon repeated use and may not be ‘relatively’ free from measurement error, while inadequate validity would lead to a measure providing results that are not a true representation of the desired constructs. It is necessary for measures to have adequate reliability and validity to ensure that results contribute valuable knowledge to the evidence base [15]. Therefore, it is essential that research builds upon past studies in the area, such as Cowin et al. [16], who psychometrically appraised professional identity measures for use with nursing students, and continues to psychometrically evaluate newly-developed professional identity measures to ensure the credibility of future findings. This will allow quantitative research to conduct comparisons between study cohorts and further the existing understanding of professional identity provided by past qualitative findings. The aims of this study were, therefore, to:

1. Identify professional identity measures that have been previously used with allied health, nursing, and medicine students enrolled in university health programs; and
2. Describe, appraise and synthesise the psychometric properties of the identified professional identity measures to make recommendations for professional identity measures to use in further quantitative studies.

## Methods

**Fig. 1:**
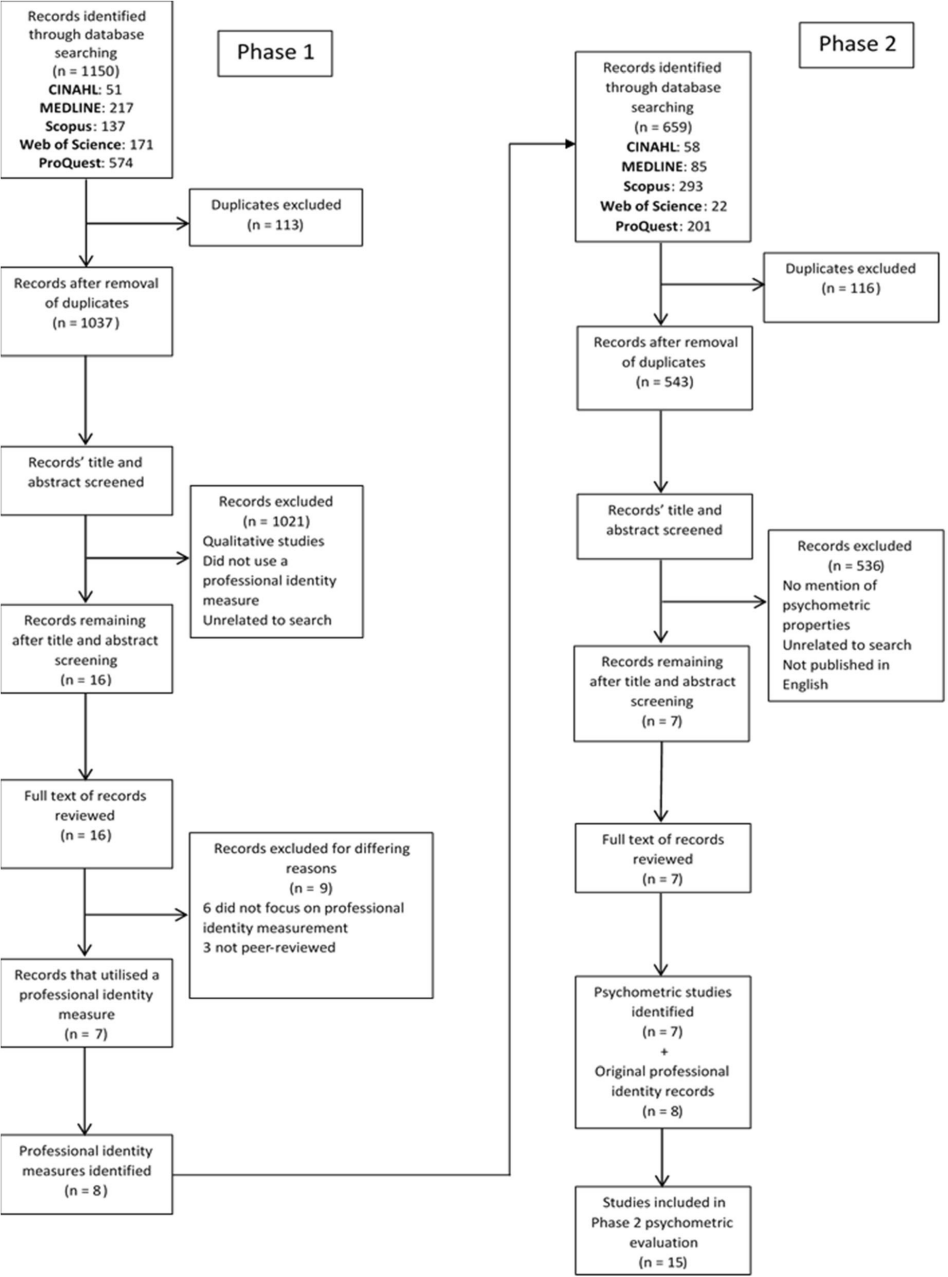
PRISMA Flow diagram of the search strategy and results

This study was conducted in two phases similar to those in the systematic review by Bialocerkowski et al. [17], who investigated the psychometric properties of outcome measures used with children and adolescents diagnosed with brachial plexus birth palsy. Phase 1 involved identifying measures that have been previously used to evaluate the professional identity of individuals studying allied health, nursing or medicine. Phase 2 involved the identification and examination of the psychometric properties for each of the identified professional identity measures (Fig. 1). All steps in both phases were conducted independently by the primary researcher (JM) and verified by another research (either AB or MM). Where discrepancies occurred, consensus was gained through discussion with the entire research team.

### Phase one

#### Databases and search strategy

A comprehensive search of five databases (CINAHL, MEDLINE, Scopus, ProQuest and Web of Science) was conducted in November, 2017. A standardised search strategy was developed for each of database utilising the Boolean operators “AND” and “OR”. Four main terms were used for the search strategy: “health”, “student”, “professional identity” and “measure”. The allied health professions included in the search strategy were selected based on the professions associated with the Allied Health Professions Australia organisation [18]. Nursing and medicine were included as exploratory searches revealed a large volume of evidence on professional identity in those professions. Further, synonyms for the terms “student”, “professional identity” and “measure” were identified and included in the search strategy. All searches were restricted to “English” for language and “journal articles” for type of publication. No restriction was placed on date of publication. Filters were used for the searches on the ProQuest (“health services” OR “health education”) and Scopus (“health professions”) databases to ensure that hits were specific to health. Table 1 provides an example of the search strategy used.

**Table 1:** Example phase 1 search strategy

Database	CINAHL
Search Terms	1, “professional identity” OR “professional confidence” OR “self-identification” *AND* 2. “allied health” OR “occupational therapy” OR physiotherapy OR “physical therapy” OR audiology OR “clinical psychology” OR “music therapy” OR dietetics OR orthotics OR prosthetics OR pharmacy OR podiatry OR “radiation therapy” OR radiography OR “social work” OR “speech pathology” OR “speech therapy” OR nursing OR health OR practitioner OR medicine *AND* 3. student OR university OR “professional education” *AND* 4. measure OR instrument OR tool OR metric OR scale OR assessment 5. Limit language to “English”

### Selection criteria

To be included for full article review, a priori criteria needed to be met. Articles were required to be quantitative studies focused on professional identity, which used a standardised measure that had been specifically developed for the evaluation of professional identity in the health professions included. For each of the articles that met these criteria, the journal in which they were published, was searched using UlrichsWeb to ensure that only peer-reviewed articles were included in this review. If it was unclear whether an article met the priori criteria, the full text was reviewed to finalise the decision.

### Selection of studies

Following the completion of the database searches, duplicates in the results were removed. The titles and abstracts of the remaining articles were screened according to the selection criteria for full article review. Each article was then reviewed to identify measures that had been used to evaluate professional identity with student health professionals. A list of the professional identity measure names was developed for Phase 2 of the study.

### Phase two

To identify evidence of their psychometric properties, the names of each professional identity measure identified in Phase 1 were searched in five databases (CINAHL, MEDLINE, Scopus, ProQuest and Web of Science). The date range of each search was limited to the date of publication referring to each measure. To be included articles had to include investigation at least one psychometric characteristic of at least one professional identity measure and be published in the English language. Following the searches, duplicates were removed. The titles and abstracts of the remaining articles were screened to identify articles which detailed psychometric properties of the professional identity measures.

Prior to critical appraisal of the psychometric evidence, descriptive information, including details regarding the study sample and details regarding the outcome measure (e.g. number of items and subscales), was extracted from each of the studies. Then, data were extracted regarding the measure’s psychometric properties, specifically test-retest reliability, internal consistency, content, structural, and cross cultural validity. The Consensus-based Standards for the Selection of Health Measurement Instruments (COSMIN) checklist [14] was utilised to assess the methodological quality of the psychometric evidence underpinning each measure. The COSMIN checklist includes twelve items: two items to assess general requirements of a study, nine items evaluating methodological qualities and psychometric properties, one item assessing the interpretability of the measure. Following the extraction of these data, they were synthesised narratively.

## Results

The study selection process and search results for both phases are depicted in Fig. 1. From the Phase 1 search strategy, 16 articles met the inclusion criteria for full-text review. Following review, eight professional identity measures were identified from nine articles: Clarity of Professional Identity [19], Macleod Clark Professional Identity Scale [1], Nurse Self Concept Questionnaire [20], Nurses’ Professional Values Scale – Revised [21], Professional Identity Five Factor Scale [22], Professional Identity Scale for Nursing Students [23], Professional Self Identity Questionnaire [24], and The Values Survey [25].

No additional professional identity measures were found during Phase 2 of the study. The Phase 2 search strategy yielded seven articles that evaluated at least one psychometric property of one or more professional identity measures. Further, all eight of the original articles that detailed the development of the identified measures were included in the Phase 2 psychometric evaluation. Therefore 15 studies were included in this review. The following section provides information on each professional identity measures and their psychometric properties. Table 2 provides a summary of the psychometric properties.

**Table 2:** Psychometric properties of professional identity measures

Measure	Reliability		Validity		
	Test-retest	Internal consistency	Content	Structural	Cross-cultural
Clarity of Professional Identity		α = 0.90^a^ [19] α = 0.76^a^ [16]	2 out of 3 supported hypotheses [19]	CFA GFI = 0.94^a^ [16]	
Macleod Clark Professional Identity scale		α = 0.79^a^ [1] α = 0.78^a^ [16] α = 0.83^a^ [26]		EFA FA = 0.46–0.73^a^ [1] PCA = 0.50–0.79^a^ [26] CFA GFI = 0.71 [16]	
Nurse Self Concept Questionnaire	ρ = 0.73–0.83* [20}	α = 0.90^a^ [20] α = 0.89^a^ [27] α = 0.95^a^ [28]	All items retained [27]	CFA GFI = 0.91^a^ [20] CFI = 0.91^a^ [27] CFI = 0.91^a^ [28] GFI = 0.87^a^ [28]	Chinese [28] Nigerian [27]
Nurses’ Professional Values Scale – Revised	r = 0.76* [21]	α = 0.92^a^ [21] α = 0.94^a^ [16] α = 0.90^a^ [29] α = 0.92^a^ [30] α = 0.93^a^ [31]	100% agreement [21] CVI = 0.90^a^ [29], 0.93^a^ [30]	EFA PCA = 0.46–0.77 [21] PCA = 0.48–0.84^a^ [29] PCA = 0.51–0.76^a^ [31] CFA CFI = 0.89 [21] NFI = 0.87 [21] GFI = 0.74 [16]	Chinese [29] Korean [31] Turkish [30]
Professional Identity Five Factor Scale		H = 0.65–0.85^a^ [22]		CFA CFI = 0.90^a^ [22]	
Professional Identity Scale for Nursing Students	r = 0.74* [23]	α = 0.83^a^ [23]	Deemed satisfactory [23]	EFA PCA = 0.48–0.69* [23]	
Professional Self Identity Questionnaire		α = 0.93^a^ [24]			
The Values Survey		α = 0.64 [25] α = 0.81^a^ [16]		EFA PCA = 0.44–0.84 [25] CFA GFI = 0.89 [16]	

*EFA* Exploratory factor analysis, *CFA* Confirmatory factor analysis, *GFI* Goodness of fit, *CFI* Comparative fit index, *CVI* Content validity index, *NFI* Normative fit index, *FA* Factor analysis, *PCA* Principal component analysis

^a^statistical result at an acceptable or better level, as reported in original publication α Cronbach’s alpha

### Clarity of professional identity

The Clarity of Professional Identity (CPI) measure was developed 2005 with the purpose of exploring how developmental network characteristics influenced professional identity over time. The measure is based upon a single-construct, and includes four items that are each rated on a 7-point Likert scale [19]. Originally, the measure was developed on 108 participants enrolled in a business program in the United States of America, However, Cowin et al. [16] has since conducted psychometric analyses of the measure using a sample of nursing students.

Two studies evaluated the psychometric properties of the CPI [16, 19]. Despite reporting a difference in the internal consistency of the measure, both studies found its internal consistency to be adequate. Dobrow and Higgins [19] found adequate content validity of the measure through the support of two out of three study hypotheses. Cowin et al. [16] found the measure to have adequate structural validity.

### Macleod Clark professional identity scale

The Macleod Clark Professional Identity Scale (MCPIS) was developed in 2006 with the aims of exploring the strength of professional identity in first year health and social care students in the United Kingdom, determining whether baseline professional identity varies between professions, and to understand the predictors of variation in baseline professional identity [1]. The MCPIS is a single-construct questionnaire with nine items, which are measured on a 5-point Likert scale. Adams et al. [1] developed the measure using data from 1254 first year health and social care students, from ten professions (Audiology, Medicine, Midwifery, Nursing, Occupational Therapy, Pharmacy, Physiotherapy, Podiatry, Radiography and Social Work).

Three studies evaluated the psychometric properties of the MCPIS [1, 16, 26]. All reported the MCPIS to have adequate internal consistency. Adams et al. [1] and Worthington et al. [26] found the measure to have adequate structural validity through exploratory factor analysis (EFA), however Cowin et al. [16] reported poor results from confirmatory factor analysis (CFA).

### Nurse self concept questionnaire

The Nurse Self Concept Questionnaire (NSCQ) was developed in 2001 as, at that time, measures of nursing professional identity were outdated and lacked psychometric evidence [20]. The NSCQ was developed using data from 506 nursing students from various Australian universities and 526 practicing nurses across Australia. The measure consists of 36 items in six subscales, with all items rated on an eight-point Likert scale. Three studies reported psychometric evidence on the NSCQ [20, 27, 28]. Cowin [20] found that the NSCQ had good internal consistency and adequate structural validity. Cross-cultural validity was supported with Nigerian [27] and Chinese [28] samples. The Nigerian study confirmed content validity through the assessment by a committee of five senior nurses. This version also was reported to have adequate internal consistency and structural validity [27]. In the Chinese study, good internal consistency, structural validity and reliability were also found [28].

### Nurses’ professional values scale – revised

The revised Nurses’ Professional Values Scale (NPVS-R) was developed in 2001 in response to changes to the 1985 nursing Code of Ethics in the United States of America [21]. The NPVS-R is a five-factor measure, incorporating 26 items which are each rated on a 5-point Likert scale. Weis and Schank [21] developed the NPVS-R based on data from 484 nursing students from 19 nursing programs and 298 practicing nurses across the USA.

Five studies evaluated the psychometric properties of the NPVS-R [16, 21, 29–31]. Two studies, other than cross-cultural studies, reported the NPVS-R to have good internal consistency [16, 21]. Weis and Schank [21] found the NPVS-R to have less than adequate structural validity through exploratory and confirmatory factor analyses, while Cowin et al. [16] reported poor structural validity. In addition, Lin et al. [29] supported the cross-cultural validity of the NPVS-R in a Chinese sample. The Chinese version of the NVPS-R was reported to have good internal consistency and adequate content and structural validity [29]. The cross-cultural validity of the Turkish [30] and Korean [31] measures was also supported with high reliability and, adequate internal consistency and structural validity.

### Professional identity five factor scale

The Professional Identity Five Factor Scale (PIFFS) was developed in 2015 to address a lack of research into professional identity development in university programs in Singapore [22]. It was developed at one Singaporean university using data from students enrolled in 36 programs across six schools [22]. The PIFFS is a five-factor measure consisting of 25 items. All items except for one, are rated on a 5-point Likert scale.

The developers of the PIPFS [22] assessed its psychometric properties. It demonstrated high levels of reliability. Construct validity was supported through sound structural validity and a “highly stable” hypothesised five-factor model [22].

### Professional identity scale for nursing students

The Professional Identity Scale for Nursing Students (PISNS) was published in 2014. It was developed to evaluate the professional identity of Chinese nursing students to aid educators to promote the development of professional identity [23]. The measure was developed based on data from 815 nursing students from varying year levels across two schools of nursing in China. The PISNS is a five-factor measure consisting of 17 items, which are each rated on a 5-point Likert scale.

The developers of the PISNS assessed its psychometric properties. It has adequate internal consistency and good test-retest reliability. A panel of four experts found the PISNS to have satisfactory content validity, and exploratory factor analysis revealed sound structural validity [23].

### Professional self identity questionnaire

The Professional Self Identity Questionnaire (PSIQ) was published in 2009. It aims to provide understanding of how different curricular features influence the development of professional identity in health and social care students [24]. The PSIQ includes nine items spanning three factors [24]. The measure was initially tested with a small sample of health and social care students, then administered to 496 medical students [24] in the United Kingdom.

The developers of the PSIQ also assessed one psychometric property [24]. Internal consistency was found to be adequate.

### The values survey

The Values Survey (TVS) was developed in 2004 to gain an understanding the underlying reasons behind Norwegian nursing students’ motivation to help others [25]. Rognstad et al. [25] developed the TVS using data from 301 s-year nursing students. The TVS consists of two factors, and a total of eight items with each item rated on a 5-point Likert-rating scale.

Two studies evaluated the psychometric properties of the TVS [16, 25]. Rognstad et al. [25] reported internal consistency slightly below the adequate level, while Cowin et al. [16] reported good internal consistency. Additionally, confirmatory factor analysis found the measure had unsatisfactory structural validity [16].

### Methodological quality of studies

Following identification of the psychometric properties for each of the measures, the COSMIN methodological quality assessment found eight of the studies to have “good” or “fair” methodological quality. This meant that the studies had reported the necessary information or had provided the reader with enough information to make conclusions about its quality according to criteria of the COSMIN checklist. One study was found to have “excellent” methodological quality [14]. Ratings are provided in Table 3.

**Table 3:** COSMIN methodological quality ratings

Psychometric study	COSMIN Rating	Measure
Adams et al. [1]	Good	Macleod Clark Professional Identity Scale
Cao et al. [28]	Fair	Nurse Self Concept Questionnaire
Cowin et al. [16]	Excellent	Clarity of Professional Identity Macleod Clark Professional Identity Scale Nurses’ Professional Values Scale – Revised The Values Survey
Cowin [20]	Good	Nurse Self Concept Questionnaire
Crossley & Vivekananda-Schmidt [24]	Fair	Professional Self Identity Questionnaire
Dobrow & Higgins [19]	Good	Clarity of Professional Identity
Geckil et al. [30]	Good	Nurses’ Professional Values Scale – Revised
Hao et al. [23]	*Fair*	Professional Identity Scale for Nursing Students
Lin et al. [29]	Good	Nurses’ Professional Values Scale – Revised
Moon et al. [31]	Fair	Nurses’ Professional Values Scale – Revised
Onyeizugbo & Nwafor [27]	Poor	Nurse Self Concept Questionnaire
Rognstad et al. [25]	Good	The Values Survey
Tan et al. [22]	*Good*	Professional Identity Five Factor Scale
Weis & Schank [21]	Good	Nurses’ Professional Values Scale – Revised
Worthington et al. [26]	Good	Macleod Clark Professional Identity Scale

## Discussion

To the best of the authors’ knowledge, no previous study has synthesised the psychometric properties of professional identity measures since 2013. Cowin et al. [16] identified five professional identity measures at that time, four of which, were included in the current study. The fifth measure was excluded from this review as it was not named and thus psychometric evidence could not be found on it during the Phase 2 search. An additional four measures were identified in the current study, which was likely the result of a comprehensive and transparent search strategy. In total, Phase 1 of this systematic review identified eight professional identity measures that had been used with university allied health, nursing, and medical students.

Phase 2 of the systematic review revealed a paucity of studies evaluating the psychometric properties of professional identity measures (*n* = 7). A lack of psychometric evaluation of measures is not uncommon. Sutton et al. [32] found that although some measures have undergone examination in the past, there is a lack of research regarding their psychometric properties which makes it difficult to determine the best measure to use. Perhaps the lack of psychometric evaluation, along with the small number of professional identity measures, is because the majority of professional identity research has used qualitative approaches [2, 9, 33]. However, although qualitative studies have provided valuable insight into professional identity development, it is critical that professional identity research begins to more frequently use psychometrically-sound quantitative approaches to further understand professional identity.

The applicability of professional identity measures for future research can be impacted by the sample population on which they were developed. The Nurses’ Professional Value Scale (Revised), The Values Survey, Professional Identity Scale for Nursing Students and Nurse Self Concept Questionnaire were specifically designed for, and evaluated on, nursing students. Their items, therefore, may not be generalizable to other health professions. This is demonstrated, for example, by one item in the Nurses’ Professional Value Scale (Revised) “Recognize role of professional nursing associations in shaping health care policy” [21]. This construct may well be more relevant to nursing compared to other healthcare professions. Thus, the validity of the Nurses’ Professional Value Scale (Revised), The Values Survey, Professional Identity Scale for Nursing Students and Nurse Self Concept Questionnaire require evaluation in a broad range of health professions, if these measures are planned to be used in allied health or medical research. In contrast, the Macleod Clark Professional Identity Scale, Professional Identity Five Factor Scale and Professional Self Identity Questionnaire have been tested on a range of health students, thus they are suitable for use in interprofessional contexts or to enable comparison between professions.

This systematic review identified a paucity of psychometric evidence for the identified professional identity measures. None of the measures had the full range of psychometric evidence, as recommended in the COSMIN checklist. Currently there is a small body of psychometric evidence available beyond the original publications of each of the professional identity measures. Reliability was reported for the Professional Identity Five Factor Scale [22] and Professional Identity Scale for Nursing Students [23] as part of their development, and both were found to be adequate. The reliability of the Nurses’ Professional Values Scale (Revised) and Nurse Self Concept Questionnaire was supported in two cross-cultural validation studies [28, 30]. Considering that reliability is the extent to which a measure is consistent and free from error, it is critical for measures to display adequate reliability to ensure the validity of their findings [17]. The current psychometric appraisal revealed that four of the identified professional identity measures have no reported reliability evaluation in the literature, which is a cause for concern. Internal consistency, which is the interrelatedness between items of a measure, is also a property of reliability [14]. In this study it was found that internal consistency evidence was available for seven of the eight measures making it the most frequently reported psychometric property. All internal consistency values were reported to be at an adequate level. It must be noted that adequate reliability is required for adequate validity [17]. Thus, the results generated from measures which are purported to have adequate validity should be interpreted with caution if no evidence of adequate reliability is found.

In addition to reliability, validity of a measure is a critical psychometric property. Structural validity was evaluated for seven of the eight measures. The original publications for the Macleod Clark Professional Identity Scale, Professional Identity Five Factor Scale, Professional Identity Scale for Nursing Students, Clarity of Professional Identity and Nurse Self Concept Questionnaire reported adequate structural validity of their respective measure, while the Nurses’ Professional Value Scale (Revised) reported an inadequate value. Cowin et al. [16] reported inadequate structural validity for the Macleod Clark Professional Identity Scale, Nurses’ Professional Value Scale (Revised) and The Values Survey [16]. These findings demonstrate inconsistencies in structural validity reporting, thus highlighting the need for future psychometric evaluation. Cross-cultural validity was explored for the Chinese, Turkish and Korean [29–31] versions of the Nurses’ Professional Values Scale (Revised), and the Nigerian and Chinese [27, 28] versions of the Nurse Self Concept Questionnaire. These studies evaluated various psychometric properties including, reliability, internal consistency, structural validity and content validity. The validity of the Nurses’ Professional Value Scale (Revised) and the Nurse Self Concept Questionnaire was supported in all tested languages.

Based on these findings, the authors were able to rate (using the categories of excellent, good, fair, poor) the methodological quality of the studies using the COSMIN checklist. Our assessment of methods suggests that the original publication studies for the Macleod Clark Professional Identity Scale, Nurses’ Professional Value Scale (Revised), Professional Identity Five Factor Scale, Clarity of Professional Identity, The Values Survey and Nurse Self Concept Questionnaire have a “good” level of methodological quality. A lack of information reported in three studies, such as details on the sample and management of missing items, resulted in the methodological quality of two studies being rated as “fair” [23, 24] and one cross-cultural validation study for the Nurse Self Concept Questionnaire being rated “poor” [27]. One psychometric study was rated “excellent” as it provided detail about each of the testing methods used [16].

Researchers are reminded to consider psychometric evidence, applicability to one’s own context, and methodological quality when selecting the most appropriate professional identity measure in the future. From the findings of this study, the Macleod Clark Professional Identity Scale appears to be the most appropriate choice of professional identity measure to use on a range of health professional students, as it has the largest body of psychometric evidence, which supports its robustness, in comparison to the other professional identity measures. The psychometric evidence available for the Nurses’ Professional Values Scale (Revised) suggests that it may be the best option for nursing professional identity research. However, the paucity of psychometric testing conducted on all identified measures means that researchers should interpret their findings with caution to ensure appropriate interpretation of the data.

### Limitations and future directions

The results of this study must be interpreted in light of its limitations. Five electronic databases were used during identify relevant evidence. It is possible that some professional identity measures may have been overlooked. This, however, is unlikely as additional measures were identified beyond those sourced by Cowin et al. [16]. Restricting the search to English language evidence may also have resulted in omitting non-English language measures. However, it was beyond the scope of this study to identify and translate evidence in languages other than English. In this study a body of heterogeneous studies met the inclusion criteria. This heterogeneity was required to summarise the body of evidence on this topic. However, this precluded meta-analysis of statistical data [34]. A narrative synthesis of data was therefore the most appropriate method to summarise the findings of this study [35]. Methodological quality of the included studies was evaluated using the COSMIN checklist, which provides a consensus-based method for evaluating psychometric evidence. Using an alternate tool to evaluate the methodological quality of the identified studies may have provided differing results, as all critical appraisal tools are arbitrary in nature [36]. Other methods could also be used to describe or facilitate the development of professional identity, such as reflective writing [11, 12], and these methods or frameworks were not included in this review.

Based on the results of this study, further psychometric evaluation of each of the identified measures is critical to the interpretation of the future study. High quality studies with methodological rigour are required to produce psychometrically-sound evidence on these professional identity measures.

## Conclusion

Professional identity is an area that is of interest to health profession educators and researchers because of the significance it has on the future practice of students in the workforce and the possible challenges that are associated with health settings. This study is the first to review professional identity measures and their psychometric properties since 2013 and is significant in demonstrating the need for further psychometric evaluation of professional identity measures. Despite the identified eight professional identity measures having some evaluation of their psychometric properties, the existing literature provides insufficient evidence for researchers to utilise any of these measures without interpreting findings with caution. Future research is necessary to further evaluate the psychometric properties of professional identity measures to ensure that the growing interest in the area is not negatively influenced by measures that have not demonstrated psychometrically-sound qualities.

## Data Availability

All data generated or analysed during this study are included in this published article.

## Abbreviations

CFA: confirmatory factor analysis
COSMIN: Consensus-based standards for the selection of health measurement instruments
CPI: Clarity of Professional Identity
EFA: exploratory factor analysis
MCPIS: Macleod Clark Professional Identity Scale
NPVS-R: Nurses’ Professional Values Scale – revised
NSCQ: Nurse Self Concept Questionnaire
PIFFS: Professional Identity Five Factor Scale
PISNS: Professional Identity Scale for Nursing Students
PSIQ: Professional Self Identity Questionnaire
TVS: The Values Survey
